# Reassortment of Avian Influenza A/H6N6 Viruses from Live Poultry Markets in Guangdong, China

**DOI:** 10.3389/fmicb.2016.00065

**Published:** 2016-02-05

**Authors:** Runyu Yuan, Lirong Zou, Yinfeng Kang, Jie Wu, Xianqiao Zeng, Jing Lu, Lijun Liang, Yingchao Song, Xin Zhang, Hanzhong Ni, Jinyan Lin, Ming Liao, Changwen Ke

**Affiliations:** ^1^Key Laboratory for Repository and Application of Pathogenic Microbiology, Research Center for Pathogens Detection Technology of Emerging Infectious Diseases, Guangdong Provincial Center for Disease Control and PreventionGuangzhou, China; ^2^World Health Organization Collaborating Centre for Surveillance, Research and Training of Emerging Infectious DiseasesGuangzhou, China; ^3^Key Laboratory of Zoonosis Prevention and Control of Guangdong, College of Veterinary Medicine, South China Agricultural UniversityGuangzhou, China; ^4^Guangdong Provincial Institute of Public HealthGuangzhou, China

**Keywords:** avian influenza virus, H6N6, live poultry market, reassortment, Guangdong

## Abstract

Since early 2013, H7N9-subtype avian influenza virus (AIV) has caused human infection in eastern China. To evaluate AIV contamination and the public risk of infection, we systematically implemented environmental sampling from live poultry markets in Guangdong Province. Through real-time polymerase chain reaction assays and next-generation sequencing, we generated full nucleotide sequences of all 10 H6N6 AIVs isolated during sampling. Focusing on sequence analyses of hemagglutinin genes of the 10 H6N6 AIVs revealed that the viruses were low pathogenic AIVs with the typical hemagglutinin cleavage site of P-Q-I-E-T-R-G. The hemagglutinin, neuraminidase, and nucleocapsid genes of nine AIVs were of ST2853-like (H6-subtype) lineage, ST192-like (N6-subtype) lineage, and HN573-like (H6-subtype) lineage, respectively; whereas the other five genes were of ST339-like (H6-subtype) lineage. However, the polymerase PB2 and nucleocapsid genes of one strain (HZ057) were of GS/GD-like (H5N1-subtype) and ST339-like lineages. Phylogenic analysis revealed that all eight genes of the 10 viruses belonged to Eurasian avian lineage. Altogether, the 10 AIVs were reassortants of different genetic groups of exchanges with the same virus subtype, thus illustrating the genetic diversity and complexity of H6N6-subtype AIVs in Guangdong Province.

## Introduction

Avian influenza viruses (AIVs) are single-stranded, negative-sense RNA viruses of the family Orthomyxoviridae that contain eight gene segments (Webster et al., 1992). Based upon the antigenicity of hemagglutinin (HA) and neuraminidase (NA), AIVs can be classified as H1–H18 and N1–N11, respectively (Webster et al., 1992; Fouchier et al., 2005; Tong et al., 2013; Zhu et al., 2013). Generally, AIVs can cause acute respiratory distress syndrome in poultry (Webster et al., 1992). Furthermore, as epidemiological and genetic analyses have revealed, during the last decade multiple AIV subtypes (H5, H6, H7, and H9) have cocirculated in various types of poultry in southern China, thereby threatening outbreaks in both poultry and humans (Cheung et al., 2007; Xu et al., 2007; Duan et al., 2008; Yu et al., 2014).

Live poultry markets (LPM) have long been regarded as potential hotbeds for AIVs, whose transmission can be facilitated there via humans' direct and indirect contact with poultry. It has been reported that LPMs have become an important source for human infection with H5N1 highly pathogenic AIV (Wan et al., 2011). Since early 2013, H7N9-subtype AIVs have emerged in eastern China and caused severe human respiratory infections (Gao et al., 2013). As of February 2015, confirmed cases of human infection caused by H7N9-subtype AIVs totaled 602, of which 227 were fatal. Furthermore, all cases were detected in China or in travelers who had visited the country (WHO, 2015). According to epidemiology analyses, LPMs contaminated with H7N9 AIVs have been predominant sources of human infection (Li et al., 2013, 2014; Yu et al., 2014). In response to all of the above evidence, environmental sampling of LPMs in China's Guangdong Province were conducted to assess the risk of human infection and to analyze the evolution of AIVs.

Surveillance of LPMs in southern China focused upon AIVs has revealed that H6-subtype is the most common AIV subtype in the region and has a more extensive range of hosts than any other subtype (Spackman et al., 2005; Munster et al., 2007). Although H6-subtype has not received much attention due to its non-lethality in poultry and mammals, recent research has reported that anti-H6 antibodies can be detected in humans (Myers et al., 2007). Because these results indicate that H6-subtype could be able to infect humans, the surveillance of the subtype in LPMs has become essential.

In this study, we isolated 10 H6N6-subtype AIVs during our environmental surveillance of LPMs in Guangdong Province. To obtain genome sequences, we used a PGM™ next-generation sequencer (Ion, American Life Technologies, Guilford, Connecticut, USA) and thereafter analyzed the molecular evolution of 10 H6N6-subtype AIVs. Ultimately, with this study we have contributed fundamental data regarding controlling AIVs as well as epidemiological and molecular evidence of their evolution in Guangdong's LPMs.

## Materials and methods

### Ethics approval statement

This study was approved by the Guangdong Provincial Center for Disease Control and Prevention (CDC) Experimental Animal Welfare Ethics Committee (permit number 2015–09).

### Virus isolation and identification

Surveillance of AIVs was conducted in LPMs in Guangdong for 12 months beginning in January 2013. Samples including poultry manure and material swabbed from defeathering machines, sewage, and chopping tools were randomly taken from LPM environments weekly. Samples were initially tested for influenza A virus with real-time polymerase chain reaction (PCR) in the local CDC. Positive influenza A samples were differentiated by HA subtype (H1–H18) via real-time PCR in local CDC laboratories and further verified by Guangdong Provincial CDC laboratories. Positive samples of H6-subtype were further tested by NA subtype (N1–N11), also with real-time PCR. In all, 10 positive samples of H6N6-subtype were purified and propagated in embryonated, 10-day-old specific-pathogen–free (SPF) chicken eggs and stored at −70°C (Table 1). Further viral subtype identification was conducted by hemagglutination inhibition assay with standard sera generated in our laboratory.

**Table 1:** **Isolation of H6N6 subtype viruses from LPM in Guangdong in 2013**.

**Virus**	**Abbreviation**	**Collection City**	**Collection Date**	**GenBank Accession No**.
A/Environment/Guangdong/GZ090/2013(H6N6)	GZ090	Guangzhou	2013.02	KT370048(PB2), KT370046(PB1), KT370037(PA), KT369980(HA), KT370017(NP), KT369998(NA), KT369990(M), KT370018(NS)
A/Environment/Guangdong/GZ523/2013(H6N6)	GZ523	Guangzhou	2013.10	KT370049(PB2), KT370045(PB1), KT370036(PA), KT369981(HA), KT370016(NP), KT369999(NA), KT369991(M), KT370019(NS)
A/Environment/Guangdong/GZ533/2013(H6N6)	GZ533	Guangzhou	2013.10	KT370050(PB2), KT370044(PB1), KT370035(PA), KT369982(HA), KT370015(NP), KT370000(NA), KT369992(M), KT370020(NS)
A/Environment/Guangdong/HZ057/2013(H6N6)	HZ057	Huizhou	2013.05	KT370051(PB2), KT370047(PB1), KT370034(PA), KT369983(HA), KT370014(NP), KT370001(NA), KT369993(M), KT370021(NS)
A/Environment/Guangdong/HZ058/2013(H6N6)	HZ058	Huizhou	2013.05	KT370052(PB2), KT370043(PB1), KT370033(PA), KT369984(HA), KT370013(NP), KT370002(NA), KT369994(M), KT370022(NS)
A/Environment/Guangdong/HZ092/2013(H6N6)	HZ092	Huizhou	2013.05	KT370053(PB2), KT370042(PB1), KT370032(PA), KT369985(HA), KT370012(NP), KT370003(NA), KT369995(M), KT370023(NS)
A/Environment/Guangdong/HZ117/2013(H6N6)	HZ117	Huizhou	2013.05	KT370054(PB2), KT370038(PB1), KT370031(PA), KT369986(HA), KT370011(NP), KT370004(NA), KT369996(M), KT370024(NS)
A/Environment/Guangdong/HZ120/2013(H6N6)	HZ120	Huizhou	2013.05	KT370055(PB2), KT370039(PB1), KT370030(PA), KT369987(HA), KT370010(NP), KT370005(NA), KT369997(M), KT370025(NS)
A/Environment/Guangdong/SW070/2013(H6N6)	SW070	Shanwei	2013.05	KT370056(PB2), KT370041(PB1), KT370029(PA), KT369978(HA), KT370009(NP), KT370006(NA), KT369988(M), KT370026(NS)
A/Environment/Guangdong/SW099/2013(H6N6)	SW099	Shanwei	2013.05	KT370057(PB2), KT370040(PB1), KT370028(PA), KT369979(HA), KT370008(NP), KT370007(NA), KT369989(M), KT370027(NS)

### Genomic sequencing

Viral RNA was first extracted from allantoic fluid with the QIAmp Viral RNA Mini Kit (Qiagen, Hilden, Germany). Reverse transcription and PCR amplification of all eight gene segments was performed using PathAmp™ FluA Preamplification Reagents (American Life Technologies, Guilford, Connecticut, United States of America). Products of PCR were purified with the Ampure XP purification kit (Beckman Coulter, Brea, CA, USA) and quantified using an Ultrospec 2000 mass spectrophotometer (Pharmacia Biotech, Uppsala, Sweden). Full genome sequences of the isolated AIVs were sequenced using the Personal Genome Machine Sequencing 200 Kit v2 (American Life Technologies) and the Ion 316 Chip V2 on an Ion PGM System to sequence.

### Sequence analysis

Full sequences of the isolated AIVs were obtained by aligning the resulting sequences against representative sequences downloaded from GenBank (Huang et al., 2010, 2012; Zhao et al., 2011). The open reading frame of each gene sequence was used for phylogenetic analyses. Neighbor-joining and maximum-likelihood trees were constructed for MEGA 6.06 with 1000 replicates (Huson et al., 2007). All branches supported by >50% replicate values were considered to be in the same group in the trees.

### Nucleotide sequence accession numbers

Nucleotide sequences obtained are available from GenBank by their accession numbers (Table 1).

## Results

### Prevalence of AIVs in LPMs

In 2013, 3235 fecal and swab samples were collected from LPMs in 22 of Guangdong Province's cities. The samples were analyzed in local CDC laboratories and Guangdong Provincial CDC. Previous research has shown that AIVs can be identified year-round, although a higher presence occurs during the winter and spring than in the summer and autumn (Huang et al., 2010). Of the 3235 samples, 579 samples (17.9%)—from 21 of the cities—were detected as positive for influenza A, whereas the constitutions of H5-subtype, H7-subtype, and H9-subtype were 6.2, 0.3, and 47.3%, respectively (Lu et al., 2014). Among these samples we also isolated 10 H6N6-subtype AIVs (Table 1).

### Phylogenetic analysis of surface genes

Phylogenetic analysis was performed to clarify the evolution of the H6N6-subtype AIVs. Complete genomes of the 10 AIVs were compared with representative H6-subtype nucleotide sequences obtained from GenBank (Huang et al., 2010, 2012; Zhao et al., 2011).

**Figure 1:**
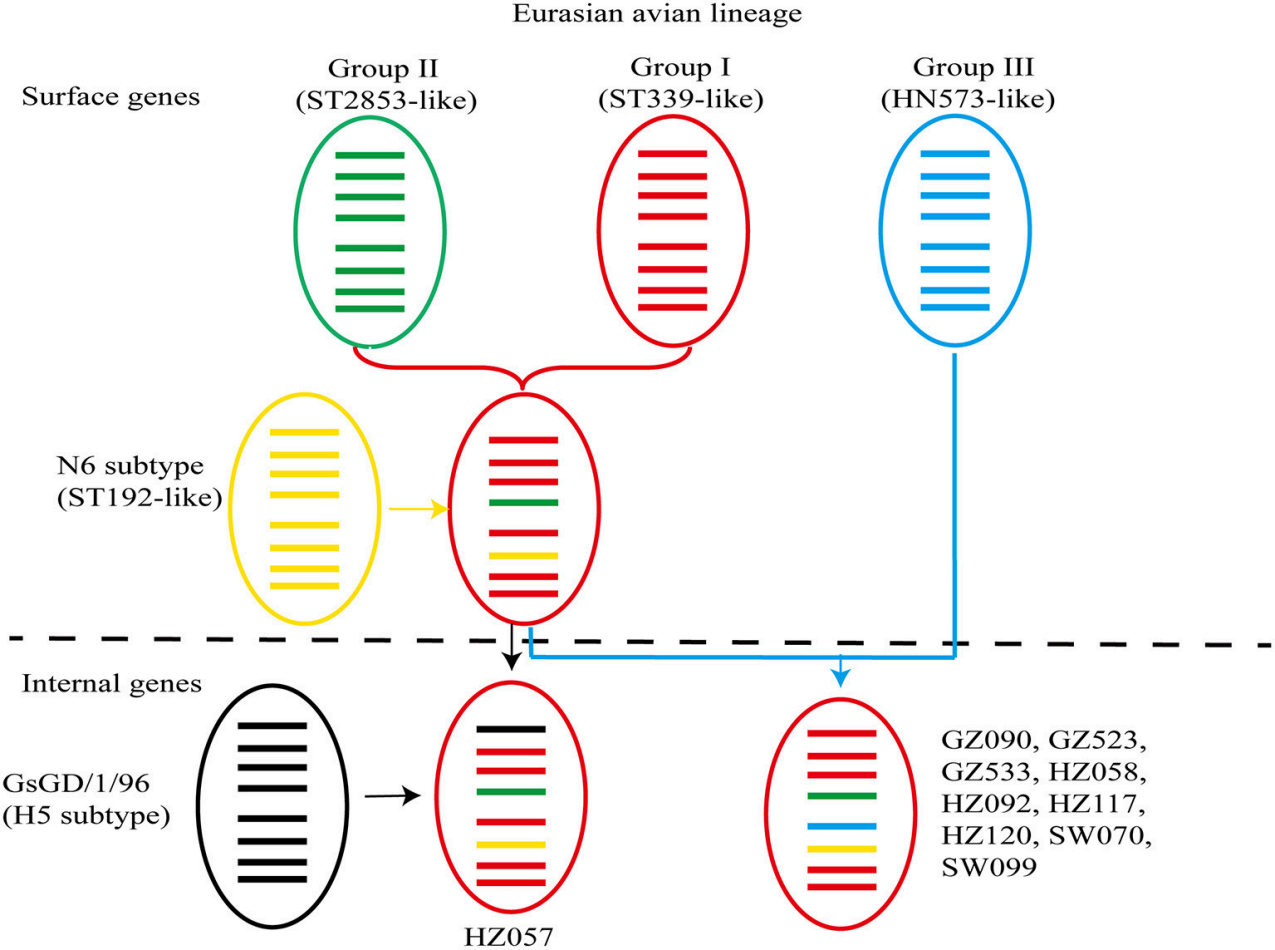
**Genetic reassortments of avian influenza virus subtype H6N6 in southern China**. From top to bottom, the eight genes in each schematic virus particle are the PB2, PB1, PA, HA, NP, NA, M, and NS genes. Genes of the same lineage appear in the same color.

**Figure 2:**
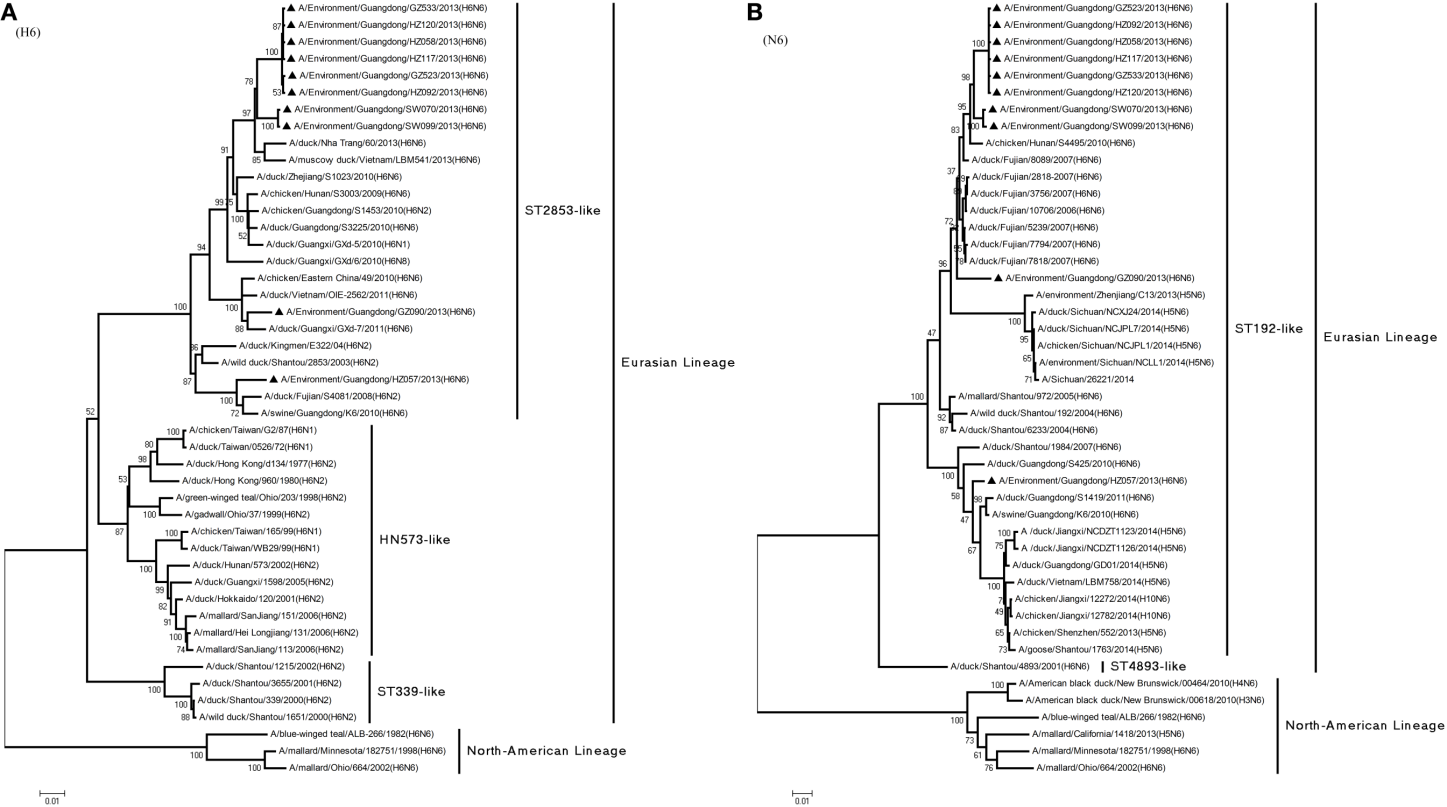
**Phylogenetic trees of the open reading frame of hemagglutinin (A) and neuraminidase (B) genes of subtype H6N6 influenza viruses isolated in Guangdong**. Viruses indicated with black triangles (▴) were characterized in this study. The trees were constructed using the neighbor-joining method of MEGA 6.06, with 1000 bootstrap trials to assign confidence to the groupings.

In a previous study of the HA gene (Huang et al., 2012), H6-subtype AIVs were isolated into two major lineages: Eurasian and North American. As shown in Figure 1, Eurasian lineage can be further divided into three groups: Group I (ST339-like), Group II (ST2853-like), and Group III (HN573-like). Figure 2A illustrates the phylogenetic tree of the HA gene, which shows all 10 viruses were ST2853-like viruses (Table 2) isolated from waterfowl in 2000–2013 (Figure 2A). Meanwhile, the 10 viruses shared 93% nucleotide identity with the HA gene of A/wild duck/Shantou/2853/2003 (H6N2).

**Table 2:** Gene composition of H6N6 subtype influenza viruses isolated from LPM in Guangdong^*^.

**Virus**	**Gene origin**							
	**HA**	**NA**	**PB2**	**PB1**	**PA**	**NP**	**M**	**NS**
GZ090	ST2853-like	ST192-like	ST339-like	ST339-like	ST339-like	HN573-like	ST339-like	ST339-like
GZ523	ST2853-like	ST192-like	ST339-like	ST339-like	ST339-like	HN573-like	ST339-like	ST339-like
GZ533	ST2853-like	ST192-like	ST339-like	ST339-like	ST339-like	HN573-like	ST339-like	ST339-like
HZ057	ST2853-like	ST192-like	Gs/GD-like; Ck/Bei-like	ST339-like	ST339-like	ST339-like	ST339-like	ST339-like
HZ058	ST2853-like	ST192-like	ST339-like	ST339-like	ST339-like	HN573-like	ST339-like	ST339-like
HZ092	ST2853-like	ST192-like	ST339-like	ST339-like	ST339-like	HN573-like	ST339-like	ST339-like
HZ117	ST2853-like	ST192-like	ST339-like	ST339-like	ST339-like	HN573-like	ST339-like	ST339-like
HZ120	ST2853-like	ST192-like	ST339-like	ST339-like	ST339-like	HN573-like	ST339-like	ST339-like
SW070	ST2853-like	ST192-like	ST339-like	ST339-like	ST339-like	HN573-like	ST339-like	ST339-like
SW099	ST2853-like	ST192-like	ST339-like	ST339-like	ST339-like	HN573-like	ST339-like	ST339-like

* *HN573-like, A/duck/Hunan/573/2002-like; ST2853-like, A/wild duck/Shantou/2853/2003-like; ST339-like, A/duck/Shantou/339/2000-like; Gs/GD-like, A/goose/Guangdong/1/96-like; Ck/Bei-like, A/Chicken/Beijing/1/94-like*.

Phylogenetic analysis of the N6-NA gene revealed its delineation into the two same major lineages—that is, Eurasian and North American (Huang et al., 2012). Eurasian lineage can be further divided into two groups: Group I (ST192-like) and Group II (ST4893-like), as shown in Figure 2B. That figure also shows that all 10 viruses were ST192-like viruses (Table 2) and shared 95.4% nucleotide identity with A/wild duck/Shantou/192/2004 (H6N6). Moreover, the NA gene of the 10 viruses shared 92–94.1% nucleotide identity with H5N6-subtype A/Sichuan/26221/2014 (H5N6), which has caused human infection and death in Sichuan Province. All of these viruses represented Eurasian lineage ST192-like branches.

### Phylogenetic analysis of internal genes

Generally, major avian-source AIVs formed the monophyletic Group I (ST339-like), which included ST339-like and ST2853-like AIVs in internal gene trees. Unlike surface gene trees, AIVs in Group II rarely formed a monophyletic clade in internal gene trees. Meanwhile, AIVs circulating in natural hosts could also form the monophyletic Group III in all internal gene trees (Figure 1).

**Figure 3:**
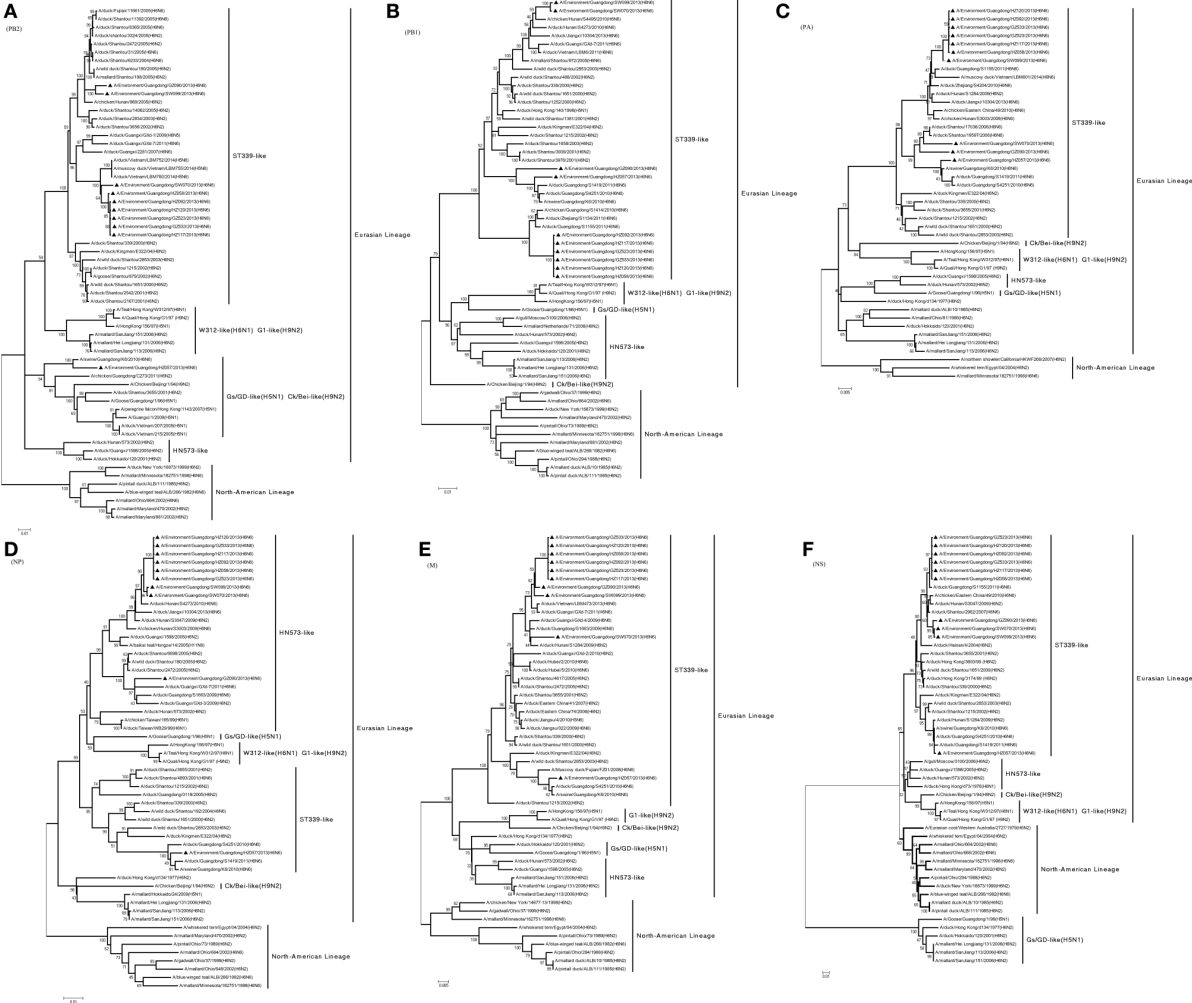
**Phylogenetic trees of the open reading frames of six internal genes of subtype H6N6 influenza A viruses isolated in Guangdong: (A) PB2, (B) PB1, (C) PA, (D) NP, (E) M, and (F) NS**. Viruses indicated with black triangles (▴) were characterized in this study. The trees were constructed using the neighbor-joining method of MEGA 6.06, with 1000 bootstrap trials to assign confidence to the groupings.

Phylogenetic analyses of internal genes showed that polymerase PB1 (PB1), polymerase PA (PA), matrix protein (M), and non-structural protein (NS) genes of all 10 viruses were of Eurasian lineage and ST339-like viruses, as well as shared 94.2–96.4, 95.3–95.5, 95.4–97.7, and 95.6–96.4% nucleotide identity with A/duck/Shantou/339/2000 (H6N2), respectively (Figures 3B,C,E,F). The polymerase PB2 (PB2) gene of nine viruses were of ST339-like lineage, although not HZ057 (Table 2), and share 94.4% nucleotide identity with the PB2 gene of A/duck/Shantou/339/2000(H6N2). As Figure 3A shows, the PB2 gene of HZ057 clustered with GS/GD-like (H5N1) and G1-like (H9N2), which caused human infection in Hong Kong in 1997. Meanwhile, the nucleocapsid (NP) gene of HZ057 belonging to ST339-like viruses did not cluster with the other nine HN573-like viruses (Figure 3D). The NP gene of the other nine viruses shared 94% nucleotide identity with A/duck/Hunan/573/2002 (H6N2) and the HZ057 NP gene shared 94.3% with the A/duck/Shantou/339/2000(H6N2). At the same time, all six internal genes of HZ057 shared 97.5–99.5% nucleotide identity with A/swine/Guangdong/K6/2010 (H6N6).

### Molecular characterization

Ten H6N6-subtype AIVs contained multiple basic amino acids (PQIETR↓G) at the cleavage site between HA1 and HA2, thereby indicating that the 10 AIVs were of low pathogenicity in domestic poultry. Amino acid residues T160, Q226, and G228 (H3 numbering) were retained in the receptor-binding pocket of HA1, thus indicating that the 10 H6N6-subtype AIVs preferred to bind to α-2,3-linked sialic acid AIV receptor (Ha et al., 2001). The 10 H6N6-subtype AIVs had six potential N-linked glycosylation sites in HA1 (Positions 26, 27, 39, 182, 306, and 311) and two in HA2 (Positions 498 and 557). By contrast, HZ058, GZ090, and SW099 exhibited an X185T mutation and lost a potential glycosylation site in their consensus amino acid sequences, whereas SW070 exhibited five potential N-linked glycosylation sites in HA1 (Positions 26, 27, 183, 307, and 312) and two in HA2 (Positions 499 and 558), as well as had an X39N mutation resulting in a loss of a potential glycosylation site in its consensus amino acid sequence.

None of the 10 AIVs demonstrated an amino acid deletion at the stalk region of the NA protein, which was related to virulence in mice. SW070 had 12 potential N-linked glycosylation sites in NA (Positions 51, 54, 67, 70, 86, 146, 171, 201, 248, 369, 402, and 434), whereas the other nine AIVs had eight potential N-linked glycosylation sites in NA (Positions 51, 54, 67, 70, 86, 146, 201, and 402). Position 274 of the NA protein (NA of GS/GD number) was Y, which suggested sensitivity to the neuraminidase inhibitor oseltamivir (Scholtissek et al., 1998; Suzuki et al., 2003).

The host specificity and virulence-related sites of PB2 were E at Position 627 and D at Position 701, thereby suggesting that the 10 viruses were avian-source ones (Massin et al., 2001; Li et al., 2005). The 198 and 317 amino acid residues of the PB1 protein were K and V, respectively, which were associated with low or no pathogenicity in mice (Katz et al., 2000). The 10 AIVs had no replacements at Positions 27, 30, or 31 of the M2 protein, thus indicating that the viruses remained sensitive to amantadine (Scholtissek et al., 1998). Though the E92D mutation was not found, the P42S mutation was found in NS1 protein of the 10 viruses, which would promote greater viral resistance to the antiviral effects of cytokines (Jiao et al., 2008; Qi et al., 2009).

## Discussion

Lying along the migratory paths of wild birds, China's Guangdong Province has several large-scale LPMs and scores of small-scale poultry farms. The warm, humid climate in Guangdong may promote the long-term survival of AIVs, thus allowing their infection of more birds and their continued proliferation. At LPMs, the mixture of different types of waterfowl and terrestrial poultry promotes interspecies transmissions from waterfowls to terrestrial poultry, all while the poultry trade of different provinces increases the diversity of AIVs and facilitates the reassortment of AIVs. Since 2013, environmental sampling programs of LPMs surveillance were implemented in Guangdong Province in order to evaluate contamination of the AIVs and to assess the public risk of human infection. During this period, we found multiple AIVs subtypes, including H5N1, H5N6, H6N6, H7N9, and H9N2, which have cocirculated in Guangdong (Lu et al., 2014). In this study, we chose 10 H6N6-subtype AIVs to further elucidate the risk of reassortment events.

H6N8-subtype AIV was first isolated from a turkey in 1963, and other H6-subtype AIVs have subsequently been isolated from wild ducks and shorebirds (Downie and Laver, 1973; Downie et al., 1973; Sharp et al., 1993). From 1975 to the early 1980s, surveillance of southern China revealed that H6-subtype AIVs occurred in waterfowl and only one H6N4-subtype AIV was isolated from chickens (Li, 1993; Matrosovich et al., 1997; Guan et al., 2000). During 2002–2008, Zhao et al. (2011) conducted an epidemiological survey in eastern China and found that subtype H6N2's isolation rate was greater than that of H6N4 and H6N6; however, after 2009, H6N6's isolation rate was greater. The H6 subtype can perpetuate in aquatic birds and domestic poultry such as chickens, ducks, and geese (Stallknecht and Shane, 1988; Huang et al., 2010; Jiao et al., 2012). Zhang et al. (2011) also isolated an H6N6-subtype AIV from swine in Guangdong (Zhang et al., 2011), thereby suggesting that the H6-subtype may have crossed the species barrier to infect mammals, including humans.

In this study, genetic evolution analysis of whole genome sequence results showed that eight gene segments of the 10 viruses shared the greatest identity with H6-subtype AIV strains isolated in Guangxi, Guangdong, and Hunan during 2005—2013. All eight fragments of the 10 viruses were of Eurasian lineage, and the genetic distance of H6N6-subtypes was remote from that of the central Chinese and North American H6N6-subtype. Our study has furthermore illustrated that the 10 viruses were reassortants of different genetic groups of the same virus subtypes (Figure 1), thus clarifying the genetic diversity and complexity of H6N6-subtype AIVs in Guangdong. It is therefore necessary to focus epidemiological and molecular research toward understanding the evolution and reassortment of AIVs in southern China.

In recent years, cases of human AIV infection have regularly occurred and thus threatened public health. H6-subtype AIVs often coexist in poultry with H5-subtype and H9-subtype, which provides opportunities for reassortment (Chin et al., 2002; Jiao et al., 2012). In 1997, a highly pathogenic H5N1-subtype AIV was proposed to be a reassortant of the AIV that derived its HA gene from A/Goose/Guangdong/1/96(H5N1) virus and its other seven genes from the H6N1 subtype A/Teal/HongKong/W312/97(H6N1) virus. This AIV caused outbreaks of disease in chickens in Hong Kong (Hoffmann et al., 2000), was later transmitted to humans, and ultimately caused six deaths among 18 infected people in 1997 (Hoffmann et al., 2000; Chin et al., 2002). In 2014, an H5N6-subtype AIV, with NA genes from subtype H6N6, caused one human death in Sichuan Province (Huang et al., 2012; China, 2014a; WHO, 2014a; Ma et al., 2015). In December 2014, Guangdong Province reported one human infection by an H5N6-subtype AIV (WHO, 2014b; OIE, 2015), whose NA gene shared with the 10 H6N6-subtype AIVs in this study both high gene homology and the same evolutionary branch. By contrast, the PB2 gene of HZ057 clustered with GS/GD-like lineage viruses that caused people to die in Hong Kong in 1997, thereby indicating that the H6N6-subtype AIV could occur naturally in reassortment with H5-subtype viruses and potentially cross the species barrier to cause human infection and even death. However, given low or no pathogenicity in poultry, H6-subtype AIVs have not received enough attention. It is therefore necessary to strengthen the surveillance of poultry and poultry environments harboring H6-subtype AIVs in LPMs and to monitor whether the subtype occurs via natural reassortment with other highly pathogenic viruses by providing external or internal genes. In terms of public health, surveillance of infection among LPM and poultry farm workers is similarly critical to alert the transmission of new reassortments from poultry to humans.

## Conclusions

In all, we have illustrated in this study that H6N6-subtype viruses isolated in LPMs constituted reassortments of different genetic groups of the same virus subtype. The genetic diversity and complexity of H6N6-subtype viruses in Guangdong Province, combined with the cocirculation of H5N1, H7N9, and H9N2 in LPMs there, indicate the high risk of the appearance of new subtypes (e.g., H5N6) and their transmission among humans.

## Author contributions

RY, YK, ML, and CK conceived and designed the experiments. RY, YS, LZ, JW, and XZ performed the experiments. RY analyzed the data. RY, LL, XZ, HN, JYL, JL, ML, and CK contributed reagents/materials/analysis tools. RY wrote the paper.

### Conflict of interest statement

The authors declare that the research was conducted in the absence of any commercial or financial relationships that could be construed as a potential conflict of interest.
